# Identification of new loci involved in the host susceptibility to *Salmonella* Typhimurium in collaborative cross mice

**DOI:** 10.1186/s12864-018-4667-0

**Published:** 2018-04-27

**Authors:** Jing Zhang, Danielle Malo, Richard Mott, Jean-Jacques Panthier, Xavier Montagutelli, Jean Jaubert

**Affiliations:** 1 Institut Pasteur, Department of Development & Stem Cell Biology, Mouse Functional Genetics, F-75015 Paris, France; 2 Centre National de la Recherche Scientifique, CNRS UMR 3738, F-75015 Paris, France; 3 McGill University Research Centre on Complex Traits, Montreal, QC, Canada; 4 University College London, UCL Genetics Institute, London, UK

**Keywords:** *Salmonella* Typhimurium, Host genetics, Bacterial infection, Collaborative cross, QTL mapping, Quantitative traits, Mouse genetics

## Abstract

**Background:**

*Salmonella* is a Gram-negative bacterium causing a wide range of clinical syndromes ranging from typhoid fever to diarrheic disease. Non-typhoidal *Salmonella* (NTS) serovars infect humans and animals, causing important health burden in the world. Susceptibility to salmonellosis varies between individuals under the control of host genes, as demonstrated by the identification of over 20 genetic loci in various mouse crosses. We have investigated the host response to *S.* Typhimurium infection in 35 Collaborative Cross (CC) strains, a genetic population which involves wild-derived strains that had not been previously assessed.

**Results:**

One hundred and forty-eight mice from 35 CC strains were challenged intravenously with 1000 colony-forming units (CFUs) of *S.* Typhimurium. Bacterial load was measured in spleen and liver at day 4 post-infection. CC strains differed significantly (*P* < 0.0001) in spleen and liver bacterial loads, while sex and age had no effect. Two significant quantitative trait loci (QTLs) on chromosomes 8 and 10 and one suggestive QTL on chromosome 1 were found for spleen bacterial load, while two suggestive QTLs on chromosomes 6 and 17 were found for liver bacterial load. These QTLs are caused by distinct allelic patterns, principally involving alleles originating from the wild-derived founders. Using sequence variations between the eight CC founder strains combined with database mining for expression in target organs and known immune phenotypes, we were able to refine the QTLs intervals and establish a list of the most promising candidate genes. Furthermore, we identified one strain, CC042/GeniUnc (CC042), as highly susceptible to *S.* Typhimurium infection.

**Conclusions:**

By exploring a broader genetic variation, the Collaborative Cross population has revealed novel loci of resistance to *Salmonella* Typhimurium. It also led to the identification of CC042 as an extremely susceptible strain.

**Electronic supplementary material:**

The online version of this article (10.1186/s12864-018-4667-0) contains supplementary material, which is available to authorized users.

## Background

*Salmonella* is a Gram-negative bacterium responsible for typhoid fever and diarrheic disease. It is one of the leading causes of food-borne infections and remains a major threat for human population [1–3]. Non-typhoidal *Salmonella* (NTS) serovars, especially *Salmonella* enterica serovar Typhimurium, infect both humans and animals, cause a significant disease burden with an estimated 93.8 million human cases and 155,000 deaths worldwide each year [4]. The variable outcome of *Salmonella* infections depends on many parameters, including the bacterial strain, environmental factors and host genetic makeup [5]. The identification of host genetic variants associated with increased resistance to the infection reveals critical mechanisms in the complex interplay between the bacteria and their host, and is instrumental to the development of effective therapies.

Infection of mice with *Salmonella* Typhimurium is widely used as an experimental model of human typhoid fever [6]. In infected mice, either orally or intravenously, there is rapid localization and replication of the bacterium in the spleen and the liver, with no clinical signs of gastroenteritis before systemic infection [7]. Laboratory mouse strains display a wide range of susceptibilities [8, 9]. C57BL/6 J (B6) strain is extremely susceptible with high spleen and liver bacterial load and death around day 5–6 post-infection, while most 129S substrains are highly resistant with low spleen and liver bacterial load and survive [10, 11]. Significant advances in understanding the host response to *Salmonella* infection have been made over the years with the identification of genes such as *Slc11a11 (Nramp1)*, *Tlr4* and *Btk,* first in the mouse model [12–15] and later in other animal species [16, 17]. SLC11A1 is expressed in the membrane of macrophages and neutrophils, and controls the replication of the bacteria by altering the *Salmonella* containing vacuole (SCV) maturation. B6 inbred strain is susceptible to *Salmonella* Typhimurium infection due to a single Gly169Asp (G169D) mutation in the predicted TM4 domain of the SLC11A1 protein, resulting in the absence of mature protein in membrane compartment. TLR4 is known by its fundamental role in bacterial outer membranes lipopolysaccharide (LPS) recognition and activation of innate immunity. BTK tyrosine-protein kinase plays a critical role in the regulation of B cell receptor signaling [5, 18].

Strategies using backcrosses or intercrosses between susceptible and resistant mouse strains to *S.* Typhimurium have been used to map various quantitative trait loci (QTLs) involved in these susceptibility differences [9, 10, 19]. QTLs confidence intervals identified in such crosses are usually broad, and identifying the causative gene(s) can be very challenging [20]. To overcome this problem, a large panel of new inbred mouse strains, namely the Collaborative Cross (CC), was developed over the last decade through a global community effort [21]. The CC strains are recombinant inbred strains derived from eight distinct founder strains that include five classical laboratory strains combined with three wild-derived strains [22, 23]. CC strains represent a genetically heterogeneous population with an even distribution of allelic variation, and a distribution of allele frequencies which closely resembles that found in human population [24]. It accounts for almost 90% of the known genetic variation present in laboratory mice originating from *M. musculus* with more than 35 million SNPs segregating between the CC founders [24]. Almost all CC have approximately equal contributions from each founder, their genome contains more recombinant events and over 90% of loci are homozygous with known genotypes [24–27].

In this study, we used the CC mouse population to identify new loci involved in the complex host response to *Salmonella* Typhimurium infection. We challenged 148 mice from 35 CC strains with 1000 CFU of *Salmonella* Typhimurium and identified two significant and one suggestive QTLs associated with spleen bacterial load, and two suggestive QTLs associated with liver bacterial load. We found that wild-derived alleles contributed largely to the effects of these QTLs. Using sequence variations of the CC founder strains combined with gene expression analysis, we identified promising candidate genes within each QTL interval. We also identified CC042/GeniUnc (CC042) as an unusually susceptible CC strain that may have further use as a model for studying *Salmonella* Typhimurium infection.

## Methods

### Animals and ethics approval

Collaborative Cross mice were purchased from the Systems Genetics Core Facility at the University of North Carolina (UNC) [27], previously generated and bred at Tel Aviv University in Israel [28], Geniad in Australia [29], and Oak Ridge National Laboratory in the US [30], and further bred and maintained at the Institut Pasteur under specific-pathogen-free (SPF) conditions. 129S2/SvPasCrl (129) and C57BL/6 J SPF mice were obtained from Charles River France and used as resistant (low bacterial loads in target organs) and susceptible (high bacterial loads) controls respectively. *Tlr4* knock-out deficient mice on B6 background (B6.129-*Tlr4*^*tm1Aki*^) were kindly provided by Jean-Marc Cavaillon (Institut Pasteur).

All animal breeding and experiments conformed to European Directive 2010/63/EU and the French regulation of February 1st, 2013 on the protection of animals used for scientific purposes. Institut Pasteur’s Animal Ethics Committee-CETEA (registered by French Research Ministry under n°89) approved experiments under numbers HA0038 and 2014–0050.

### *Salmonella* Typhimurium infection

Mice were confined in a biosafety level 3 (BSL-3) animal facility a few days prior to infection. *Salmonella* Typhimurium strain SL1344, obtained from the National Collection of Type Cultures (NCTC 13347), was used for infection. One mL of bacteria frozen culture was grown in 50 mL of Trypticase soy broth (Biorad) at 37 °C to reach the exponential phase, with an optical density at 600 nm of 0.1–0.2. The exact bacterial density was determined by plating 10^− 5^ dilutions onto tryptic soy agar. Bacterial suspension was diluted to 5000 CFU/ ml in PBS. Mice were infected by injection in the caudal vein with 1000 CFU of *Salmonella* Typhimurium in 200 μl. The infectious dose was verified following infection by serial dilutions of the inoculum plated on trypticase soy agar. Infected animals were monitored daily post infection, and body-condition scoring (score < 2) was used for clinical endpoint. A total of 148 mice from 35 CC strains, both males and females, in the age range of 7–20 weeks were tested in 18 experiments (*N* = 2 to 24 mice per experiment). B6 mice were included in every experiment and 129 mice in most of them, as susceptible and resistant controls, respectively. *Salmonella* Typhimurium strain Keller, originally obtained from Dr. Hugh Robson (Royal Victoria Hospital, Montreal, Quebec), was also used for infecting CC042/GeniUnc strain to confirm its extreme phenotype with another *Salmonella* strain.

### In vivo bacterial loads

Mice were euthanized by exposure to CO_2_ at day 4 post infection. Spleen and liver were removed aseptically, weighed, placed in 2 mL of isotonic saline and homogenized using a tissue homogenizer (T25 Ultra-Turrax, IKA). The resulting homogenate was diluted in 1× PBS and serial dilutions were plated on tryptic soy agar to determine organ bacterial load.

### In vivo LPS response

Mice were injected intraperitoneally with either 0.5 ml of PBS alone or 0.5 mL of PBS containing 100 μg of protein-free (0.008%) *Escherichia coli* K235 LPS. After 90 min, the mice were euthanized, and serum was collected by cardiac puncture. Tumor necrosis factor alpha (TNF-α) concentration in serum was measured using Mouse TNF-alpha DuoSet kit (R&D system).

### Genotyping and reconstruction of CC genome

CC strains have been previously genotyped at Wellcome Trust Centre for Human Genetics (Oxford, UK) and at UNC (Chapel Hill, USA) with several high-density arrays, including Mouse Universal Genotyping Array (MUGA and MEGA-MUGA) containing respectively 7.5 and 77.8 K SNPs [31] and Mouse Diversity Array (MDA) containing 620 K SNPs [32]. All the polymorphic SNPs homozygous in all founder strains were selected and introduced in HAPPY format using build 37 of the mouse reference genome. Each CC genome was reconstructed as a haplotype mosaic using a Hidden Markov Model (HMM) in HAPPY software [33] to estimate the probabilities of descent from each founder strain at each locus. Even though the CC mice used were nearly inbred at the time of the experiment, several strains still had ~ 10% of heterozygous genome as determined by the joint heterozygosity of obligate ancestors. Therefore, we ran in the reconstruction process the HMM under the diploid heterogeneous model mode to trace back each chromosome separately, and to allow for residual heterozygosity, averaging the reconstructions over blocks of *n* = 20 consecutive SNPs to reduce computational complexity. We set the number of generations of inbreeding at 20 as previously described [34].

### Statistical analysis

Bacterial load data analysis was performed using R statistical software. Analysis of Variance (ANOVA) was used for testing the influence of sex, age and experiment on the bacterial loads.

Mean bacterial loads were compared by one-way ANOVA and Tukey HSD test (Prism 6.0 software, GraphPad, La Jolla, CA, USA). Mean TNF-alpha responses to PBS and LPS injections were compared by two-way ANOVA and Holm-Šídák test (Prism 6.0 software, GraphPad, La Jolla, CA, USA).

### QTL mapping

QTL mapping was performed under R statistical software (release version 3.2.0) with the happy.hbrem package [33, 35]. Individual phenotypic data were transformed to account for experiment effect: i) first, data was fitted with the mean load of B6 control mice tested in each experiment using a linear regression model; ii) second, the residuals from the model were extracted and data were normalised; iii) third, mean values of normalised residuals for each strain were used for QTL mapping. The presence of a QTL was tested by an ANOVA test by comparing the fit of the genetic model with the null hypothesis. QTL mapping with CC strains consists of eight-way haplotype linear regression with additive model as previously described [34]. The number of observations for each strain was used to weight the strain averaged value in the regression analysis. Significance is reported as the -log_10_(P) value as computed by the R ANOVA function. Genome-wide significance (E < 0.5, E < 0.1 and E < 0.05) was estimated by permuting the CC strains (1000 tests). QTLs confidence intervals were defined using a 1.5 log_10_-drop method.

### Founder effect estimation, merge analysis and candidate genes selection

Founder contributions for each trait analyzed were determined by hierarchical Bayesian random-effects model using the happy.hbrem package. To distinguish the contribution of each founder, WSB/EiJ estimate was set to 0 and its effect represented by the mean value between all founders, while other founder effects were presented as the difference from the effect of WSB. To identify potentially causal SNPs in each QTL interval, we used the merge analysis [36]. Most SNPs have only two alleles, thus we merged the eight CC founders into (typically) two groups according to their allelic variation based on sequence data in the founder strains. Instead of testing for phenotypic differences between all eight founders to test for a QTL in a given interval, differences are tested between the groups of merged founders. The reduction in the dimension of the test results in increased merge logP-values compared with interval logP-values in variants responsible for the QTL. Merge analysis provides an efficient tool to prioritize SNPs within QTL intervals.

To obtain information on genes’ annotation, expression, GO function or known mutation phenotype, several public databases were used, including Mouse Genome Informatics [37], TxDb.Mmusculus.UCSC.mm9.knownGene R package [38], Immunological Genome Gene Skyline [39], ENSEMBL [40], and International Mouse Phenotyping Consortium [41].

## Results

### Diversity in response to *S.* Typhimurium infection

To explore the influence of genetic diversity of CC mice on their susceptibility to *Salmonella* Typhimurium infection, we infected groups of mice from 35 CC strains and measured spleen and liver bacterial loads at day 4 post-infection. To assess reproducibility and normalize data across experiments, B6 were included in all experiments and 129 in most of them, as reference susceptible and resistant strains, respectively.

Since the study was carried out in 18 successive experiments with mice from both sexes and at different ages, we firstly evaluated sex, age and experiment effects. No consistent significant differences were found between males and females (*P* = 0.03 and 0.89 for spleen and liver respectively, threshold *P* = 0.025 after Bonferroni correction), nor between mice of different ages (*P* = 0.46 and 0.14 for spleen and liver). However, variations between experiments were statistically significant (*P* < 2.2 × 10^− 16^ for both spleen and liver). To take into account the variability across experiments, we used the mean value of B6 mice tested in each experiment to adjust CC individual data to perform QTL mapping.

**Fig. 1:**
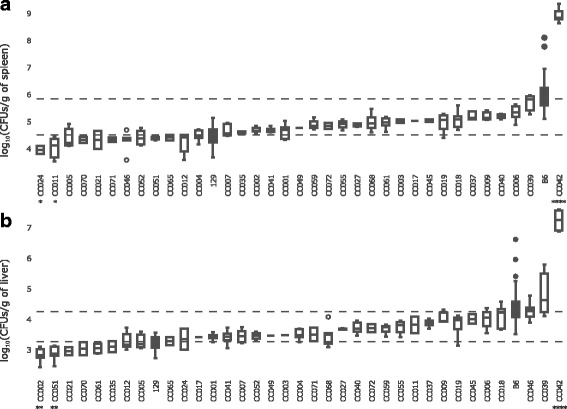
Bacterial loads in CC, B6 and 129 control strains at day 4 post-infection with *Salmonella* Typhimurium. Groups of 7–20 weeks old mice for 35 CC strains (open), B6 and 129 (closed), were inoculated with 10^3^ CFUs of *Salmonella* Typhimurium strain SL1344, and euthanized 4 days after infection for organ bacterial load assessment. Results are presented as the log_10_(CFUs/g of tissue) in spleen (**a**) and in liver (**b**). Phenotype of each strain is shown as boxes and whisker plots. The bottom, medium and top band of boxes correspond to the first, second and third quartiles. Individual grey dots represent outlier animals. The minimum and maximum bacterial load values for each strain are represented by the ends of whiskers. Dashed lines indicate the median values of B6 (high) and 129 (low) reference strains. CC042 showed extremely high level of bacteria in both organs compared to B6 mice, while CC011/Unc and CC024/GeniUnc had 3 to 4-fold lower spleen CFU counts than 129 mice; CC002/Unc and CC051/TauUnc had 3-fold lower liver CFUs counts than 129 mice. Mean bacterial loads between extreme CC strains and control resistant or susceptible strains were compared by one-way ANOVA test. Asterisks indicate respective *P*-value of *P* < 0.1(*), *P* < 0.01 (**), *P* < 0.001 (***), *P* < 0.0001 (****)

In spleen, B6 susceptible strain had a median bacterial load of 1 × 10^5.8^ CFUs/g while 129 resistant strain had a median of 1 × 10^4.5^ CFUs/g, confirming the difference reported in the literature. The 35 CC strains tested (148 mice in 18 experiments, summary in Additional file 1: Table S1) showed a wide range of responses with median CFUs/g ranging from 10^3.9^ to 10^8.8^ (Fig. 1a). Differences between CC strains in splenic bacterial loads were highly significant (*P* < 2.2 × 10^− 16^). In liver, B6 had a median bacterial load of 1 × 10^4.2^ CFUs/g and 129 a median of 1 × 10^3.2^ CFUs/g. The 35 CC strains tested had median CFUs/g ranging from 10^2.8^ to 10^7.2^ (Fig. 1b). The differences between CC strains were highly significant (P < 2.2 × 10^− 16^).

The majority of CC strains showed similar bacterial loads in their spleen and liver, with a strong positive correlation between the two traits (Pearson R^2^ = 0.88). Interestingly some CC strains showed extreme phenotypes: CC042 mice had in both organs 1000-fold higher CFU levels than B6 susceptible control mice (*P* < 2.6 × 10^− 4^), while CC011/Unc and CC024/GeniUnc had 3 to 4-fold lower spleen CFU levels than 129 resistant control mice (*P* < 0.08), CC002/Unc and CC051/TauUnc had 3-fold lower liver CFUs levels than resistant control mice (*P* = 0.001 and *P* = 0.006, respectively). CC046/Unc had spleen CFUs similar to 129 strain while liver CFUs were as high as B6.

### QTL mapping reveals five susceptibility loci

**Fig. 2:**
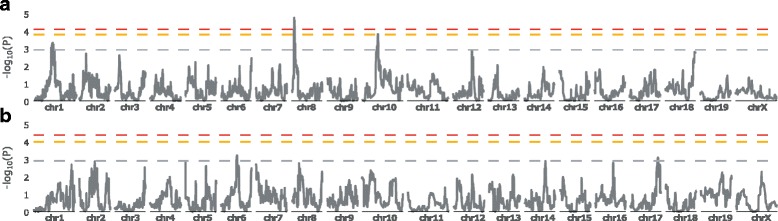
QTLs associated with bacterial loads in spleen and liver after *S.* Typhimurium infection in CC strains. Genome-wide associations for bacterial loads in spleen (**a**) and liver (**b**) in 35 CC strains. X-axis: genome location; Y-axis: –log_10_(P) values of the test of association between genotype and phenotype. Genome-wide thresholds of association at E < 0.5, E < 0.1 and E < 0.05 significance levels are indicated respectively by horizontal gray, orange and red lines. (**a**) Spleen bacterial load thresholds respectively at –log_10_(P) = 2.9, 3.8 and 4.2. (**b**) Liver bacterial load thresholds respectively at –log_10_(P) = 2.9, 4.0 and 4.3

To identify host genes controlling the variation in susceptibility to *Salmonella* Typhimurium in CC strains, we tested the association between the median organ bacterial load of the 35 CC strains and the founder haplotype probabilities based on the genotyping data of the CC strains. Five QTLs were mapped at genome-wide E < 0.5, associated with spleen or liver bacterial load and will be referred to as *Salmonella Typhimurium susceptibility loci-1* (*Stsl1*) to *Stsl5*, by order of decreasing statistical significance. QTL confidence intervals were established using 1.5 log10-drop. Figure 2 and Table 1 summarize the significant level, peak position, as well as interval width of each QTL.

**Table 1:** QTLs associated with host bacterial loads in spleen and liver post S. Typhimurium infection

QTL	Organ	Chr	-log10(P)	Peak position (Mb)	Sig. level	Interval (Mb)	Width (Mb)	All type genes	Protein coding genes	RNA genes	Unclassified genes	Unclassified non-coding RNA genes
*Stsl1*	spleen	8	4.7	12.5	0.95	11.3–17.0	5.7	144	50	68	23	3
*Stsl2*	spleen	10	3.8	52.3	0.9	46.4–54.0	7.6	62	24	30	6	2
*Stsl3a*	spleen	1	3.3	83.9	0.5	77.5–95.9	21.8	503	226	200	66	11
*Stsl3b*	spleen	1	3.0	79.2	0.5	74.1–81.8						
*Stsl4*	liver	6	3.2	81.2	0.5	77.1–90.0	12.9	336	142	128	59	7
*Stsl5*	liver	17	3.1	84.8	0.5	80.5–91.1	10.6	177	61	94	12	10

QTL nomenclature, target organ, chromosome (Chr), negative log10 p-value (−log10(P)), peak position (in Mb, build mm9), genome-wide significance level (Sig. level), QTL position and Width (in Mb, build mm9) are given. Numbers of total genes, protein coding genes, RNA genes, unclassified genes and unclassified non-coding RNA genes within each QTL interval are also presented

Three QTLs on chromosomes (Chr) 1, 8 and 10 were associated with spleen bacterial load at genome-wide E < 0.5 (Fig. 2a). *Stsl1* (11.3–17 Mb) and *Stsl2* (46.4–54 Mb) are on Chr 8 and 10, with respective -log_10_(P) values of 4.7 (E < 0.05) at 12.5 Mb and 3.8 (E < 0.1) at 52.3 Mb. On Chr 1, we identified two closely linked suggestive QTLs, with respective -log_10_(P) of 3.28 (E < 0.5) at 83.9 Mb and 3.02 (E < 0.5) at 79.2 Mb. Their confidence intervals are 77.5–95.9 Mb and 74.1–81.8 Mb respectively, and partly overlap. We named them *Stsl3a* and *Stsl3b* since we can’t conclude whether they are one or two distinct QTLs. For liver bacterial load, two QTLs were mapped at genome-wide E < 0.5 (Fig. 2b). *Stsl4* (77.1–90 Mb) and *Stsl5* (80.5–91.1 Mb) are on Chr 6 and 17 with respective -log_10_(P) of 3.2 (E < 0.5) and 3.1 (E < 0.5).

The strain CC042 shows an exceptionally susceptible phenotype in our study. Since we suspected that extreme trait values could have a strong weight on QTL identification, we re-ran QTL mapping analysis without CC042. However, since the results were essentially similar (data no shown), we included all 35 strains in the analysis.

We wondered whether the same QTLs could have been identified with a smaller number of CC strains. To this end, we ran QTL mapping on random subsets of 15, 20, 25, 30 or 34 strains (500 permutations for each subsets of strains, and 35 permutations for the 34 subsets) and we computed the frequency at which *Stsl1* and *Stsl2* could be identified, and at which genome-wide significance (see Additional file 2: Figure S1). Subsets of less than 20 strains were almost never able to detect either of the two QTLs. Subsets of 25 and 30 strains detected *Stsl1* in 45% and 90% of cases, while *Stsl2* was found in 26% and 63% of cases respectively. Subsets of 34 strains detected *Stsl1* in 100% of cases and *Stsl2* in 97% of cases. This suggests that these two QTLs are robust.

### Estimation of founder effect shows complexity

The presence of a QTL implies a contrast in mean trait values between mice carrying different haplotypes at the locus. To understand the underlying cause of the identified QTLs, we estimated for each of them the effects of the eight founder haplotypes across the QTL interval and at the peak location.

**Fig. 3:**
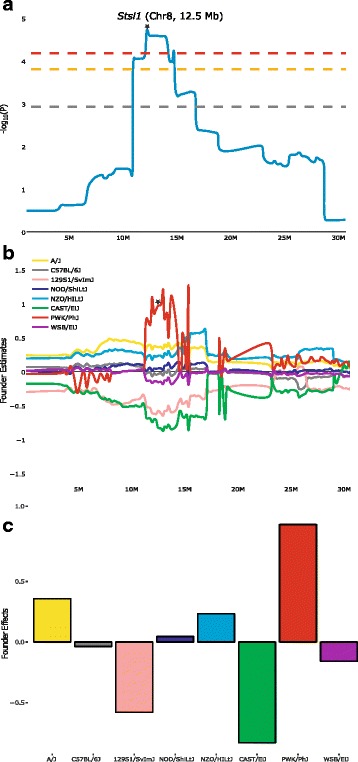
Founder contributions and haplotype around *Stsl1* QTL on Chr 8. (**a**) Genome scan magnification for *Stsl1* QTL region (0–30 Mb on Chr 8). The mouse genome location is on the X-axis and significance (−log_10_(P)) values on the Y-axis, with genome-wide thresholds of association at E < 0.5, E < 0.1 and E < 0.05 levels indicated respectively by the gray, orange and red lines. Peak location (maximum value of –log_10_(P)) is marked by a star. (**b**) Founder contributions in the same magnified region. The peak location is marked by a star. Each of the 8 founders is in a different color. The mouse genome location is on the X-axis and Y-axis shows the founder estimated effect on splenic bacterial load after *S.* Typhimurium infection. (**c**) Founder contributions at *Stsl1* QTL peak (12.5 Mb). X-axis shows the different founder strains. Y-axis shows the estimated founder effect. *Stsl1* QTL is caused by contrast between 129S1SvlmJ, CAST/EiJ (lower values) and PWK/PhJ (higher values)

**Fig. 4:**
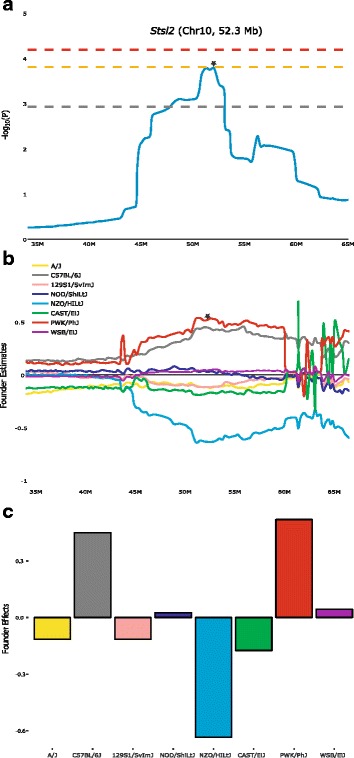
Founder contributions and haplotype around *Stsl2* QTL on Chr 10. (**a**) Genome scan magnification for *Stsl2* QTL region (35–65 Mb on Chr 10). The mouse genome location is on the X-axis and significance (−log_10_(P)) values on the Y-axis, with genome-wide thresholds of association at E < 0.5, E < 0.1 and E < 0.05 levels indicated respectively by the gray, orange and red lines. Peak location (maximum value of –log_10_(P)) is marked by a star. (**b**) Founder contributions in the same magnified region. The peak location is marked by a star. Each of the 8 founders is in a different color. The mouse genome location is on the X-axis and Y-axis shows the founder estimated effect on splenic bacterial load after *S.* Typhimurium infection. (**c**) Founder contributions at *Stsl2* QTL peak (52.3 Mb). X-axis shows the different founder strains. Y-axis shows the estimated founder effect. *Stsl2* QTL is caused by contrast between B6, PWK/PhJ (lower values) and NZO/HiLtJ (higher values)

*Stsl1* is localized on Chr 8 with a peak location at 12.5 Mb (Fig. 3a). The founder contributions, calculated across the critical interval (Fig. 3b) and at the peak location (Fig. 3c), indicates a contrast between PWK/PhJ (higher spleen bacterial loads) versus 129S1/SvImJ and CAST/EiJ strains (lower values). *Stsl2* is localized on Chr 10 with a peak at 52.3 Mb (Fig. 4a). The founder contribution indicates a contrast between PWK/PhJ and B6 (higher) versus NZO/HILtJ (lower, Fig. 4b and c). *Stsl3* is composed of two distinct peaks on Chr 1, respectively *Stsl3b* at 79.2 Mb and *Stsl3a* at 83.9 Mb (see Additional file 3: Figure S2). The founder contribution is different between the two peaks. For *Stsl3b*, it indicates a contrast between 129S1/SvImJ and B6 (higher) versus PWK/PhJ (lower). For *Stsl3a*, it indicates a contrast between B6 (higher) versus the others. *Stsl4* peak location is at 81.2 Mb on Chr 6 (see Additional file 4: Figure S3). The founder contribution is a contrast between NZO/HILtJ and PWK/PhJ (higher) versus B6 (lower). *Stsl5* peak location is at 84.8 Mb on Chr 17 (see Additional file 5: Figure S4). The founder contribution mainly shows a contrast between B6 (higher) versus NOD/ShiLtJ (lower). In conclusion, the different QTLs are caused by markedly distinct patterns of contrasts between founders, with multi-allelic variations involved.

### Association analysis of sequence variants and candidate genes

The confidence intervals of the QTLs we mapped encompass too many genes to make assumptions on the most likely candidates (Table 1). In particular, a total of 144 genes and 62 genes were identified from public databases within the most significant QTLs, *Stsl1* and *Stsl2,* respectively. In order to prioritize genes within these two intervals, we performed merge analysis [34, 36, 42] on SNP variants within these QTLs and combined the results with gene expression data.

Merge analysis reduces the dimensionality of statistical tests by merging data from strains which share the same allele at a given SNP. If a QTL is caused by a single variant with a particular strain distribution pattern (SDP) among the founders, those nearby SNPs with the same SDP will have higher logP-values than in an 8-way haplotype linear model, as a result from reduced dimensionality of the test.

**Fig. 5:**
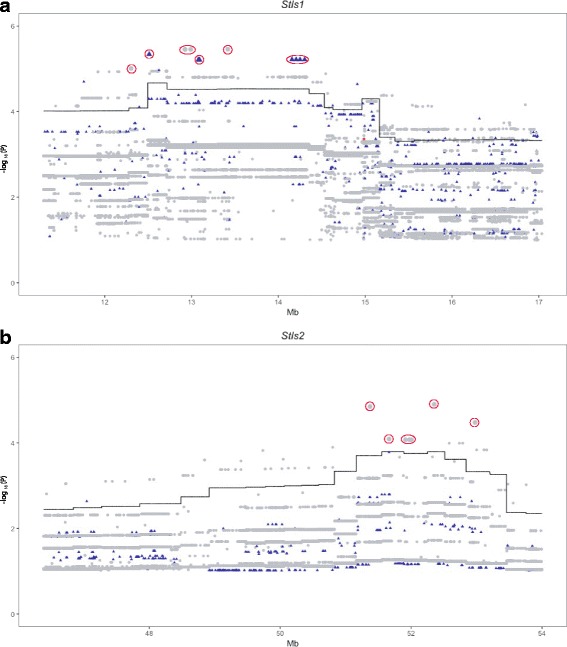
Merge analysis of sequence variants around *Stsl1* and *Stsl2* interval. The X-axis is genome location; the Y-axis is the –log_10_(P) of the test of association between locus and bacterial load. Only the critical interval for each locus is shown. The continuous black line is the genome scan result in Fig. 3. The dots correspond to the results of the merge analysis. Biallelic SNPs are in gray; Triallelic SNPs are in blue; Variants with more than three alleles are in red. The larger dots circled in red correspond to SNPs with the most significant merge –log10(P). All SNPs with merge –log_10_(P) < 1 are not shown. **a**) *Stsl1* QTL on Chr 8. **b**) *Stsl2* QTL on Chr 10

As expected, we found a fraction of SNPs with higher merged logP-values (dots and triangles in Fig. 5) than interval mapping logP-value (continuous line) near *Stsl1* and S*tsl2*, enabling to filter out the majority of SNPs (Fig. 5). For *Stsl1*, both multi-allelic (triangles) and bi-allelic (dots) variants were found among the significant SNPs, which likely reflects the complexity of the predicted founder contributions. For *Stsl2*, only bi-allelic SNPs (dots) were found among the significant SNPs. Therefore, merge analysis allowed us to reduce the number of candidate genes to 60 genes for *Stsl1* (see Additional file 6: Table S2) and 11 genes for *Stsl2* (see Additional file 7: Table S3), with only 32 genes and 6 genes possessing significant merge SNPs nearby (highlighted in red circles in Fig. 5) for *Stsl1* and *Stsl2*, respectively. To further prioritize our candidate gene list, we prioritized genes expressed by immune cells. We used MGI, ENSEMBL, ImmGen and IMPC to evaluate gene expression levels in immune cells, ontology terms and, known functions or phenotypes. As a result of this combined analysis, *Stsl1* interval most promising candidates are *Cul4a*, *Lamp1*, *Mcf2l* and *Pcid2*, which are expressed in immune cells along with significant SNPs nearby. *Stsl2* interval contains 4 out of 11 genes expressed in immune cells, while the most promising gene *Slc35f1* with significant SNPs nearby is not reported to be expressed in splenic immune cells.

### *Tlr4* is functional in the CC042 extreme susceptible strain

CC042 strain showed extreme susceptibility to *S.* Typhimurium with a 1000-fold higher organ bacterial load than in B6. CC042 has inherited a B6 susceptible allele at *Slc11a1* locus which contributes to its phenotype. However, additional alleles are required to explain its enhanced susceptibility. We observed that CC042 has also inherited a wild-derived PWK/PhJ haplotype at the *Tlr4* locus, which contains several missense mutations (Sanger mouse SNP viewer [43]). *Tlr4*-deficient mice show very high susceptibility to *Salmonella* infection due to a defective response to LPS [44, 45]. Although we did not identify any QTL in the Chr 4 region which contains *Tlr4*, we wondered whether the CC042 allele of *Tlr4* was functional.

**Fig. 6:**
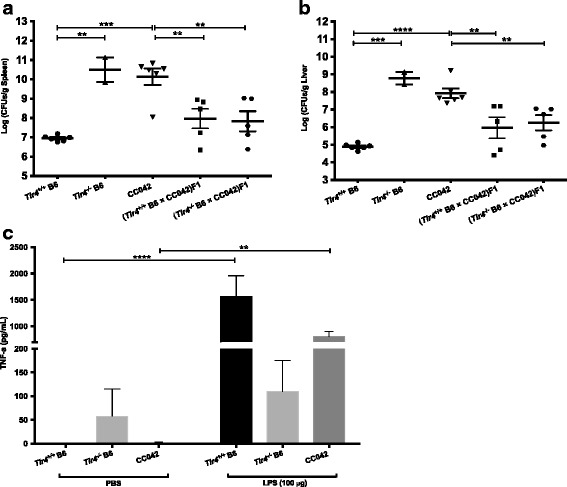
*Tlr4* gene functional study in CC042/GeniUnc. Splenic (**a**) and liver (**b**) bacterial loads at day4 p.i. in *Tlr4*^*+/+*^ B6 (*n* = 6), *Tlr4*^*−/−*^ B6 (*n* = 2), CC042 (*n* = 6), (*Tlr4*^*+/+*^ B6 x CC042)F1 (*n* = 5) and (*Tlr4*^*−/−*^ B6 x CC042)F1 (*n* = 5) mice infected with 10^3^ CFUs of *S.* Typhimurium (cumulative results from 2 distinct experiments). The TNF-alpha levels production at 90 min post-mock-PBS injection or 100 μg LPS injection (**c**) in *Tlr4*^*+/+*^ B6 (*n* = 4 and 4), *Tlr4*^*−/−*^ B6 (n = 4 and 4) and CC042 (n = 4 and 5) mice. The values are means and error bars present standard error of mean. Mean bacterial loads (**a**) and (**b**) were compared by one-way ANOVA and Tukey HSD test. Mean TNF-alpha values (**c**) were compared by two-way ANOVA and Holm-Šídák test. Asterisks indicate respective P-value of *P* < 0.1 (*), *P* < 0.01 (**), *P* < 0.001 (***), *P* < 0.0001 (****)

We first infected CC042, (*Tlr4*^*+/+*^ B6 × CC042)F1 and (*Tlr4*^*−/−*^ B6 × CC042)F1 mice with *S.* Typhimurium and compared organ bacterial loads 4 days later. Wild-type *Tlr4*^*+/+*^ B6 mice and mutant knock-out *Tlr4*^*−/−*^ B6 mice were included as positive and negative controls, respectively. Figure 6a shows that *Tlr4*^*−/−*^ B6 mice had mean bacterial loads as high as 10^10^ CFUs in the spleen, > 1000-fold higher than in *Tlr4*^*+/+*^ B6 mice. Very high bacterial loads were also observed in the spleen of CC042 mice. By contrast, (*Tlr4*^*+/+*^ B6 × CC042)F1 and (*Tlr4*^*−/−*^ B6 × CC042)F1 mice had lower bacterial loads (10^8^ CFUs) in the spleen, which were not statistically different from that measured in *Tlr4*^*+/+*^ B6 mice. In the liver, *Tlr4*^*−/−*^ B6 and CC042 mice had bacterial loads higher than 10^8^ CFUs, that is > 1000-fold higher than the bacterial loads measured in *Tlr4*^*+/+*^ B6 mice. The bacterial loads in the liver of (*Tlr4*^*+/+*^ B6 × CC042)F1 and (*Tlr4*^*−/−*^ B6 × CC042)F1 mice were both 10^6^ CFUs, 10-fold higher than in *Tlr4*^*+/+*^ B6 mice (Fig. 6b). Since *Tlr4* KO is fully recessive, we conclude that the *Tlr4* allele is functional in CC042.

LPS is a major component of the outer membranes of Gram-negative bacteria, including *Salmonella* Typhimurium. TLR4-mediated LPS response results in the release of various pro-inflammatory cytokines, including TNF-α [46]. We also investigated the in vivo response of the CC042 strain to LPS. Wild-type *Tlr4*^*+/+*^ B6 and mutant *Tlr4*^*−/−*^ B6 mice were used as positive and negative controls respectively. Mice were injected with PBS alone or PBS containing 100 μg LPS and their TNF-α serum concentration measured at 90 min post-injection. Figure 6c shows that all mice treated with PBS alone failed to release TNF-α and its levels were < 58 pg/ml. Wild-type *Tlr4*^*+/+*^ B6 mice injected with LPS produced significant levels of TNF-α (1561 pg/ml) compared to PBS-treated *Tlr4*^*+/+*^ B6 animals (ANOVA test, *p* < 0.0001). By contrast, *Tlr4*^*−/−*^ B6 mice injected with LPS failed to produce TNF-α (110 pg/ml) and showed no difference with PBS-treated animals. Interestingly, CC042 mice had a significant increase in TNF-α levels compared to PBS-injected mice (803 pg/ml, *p* = 0.009). These results confirm that *Tlr4* allele is functional in the CC042 strain.

## Discussion

In this study, we have used Collaborative Cross mice to explore a genetic diversity larger than in previous studies, which could result in identifying novel phenotypes and host genes controlling susceptibility to *Salmonella* infection.

Compared to classical laboratory inbred strains, the 35 CC strains tested exhibited wider range of bacterial loads in the spleen and liver target organs at day 4 post infection. Interestingly, some CC strains showed phenotypes beyond the previously reported range, with four strains (CC011/Unc, CC024/GeniUnc, CC002/Unc and CC051/TauUnc) having 3 to 4-fold lower CFUs in spleen or liver than 129 resistant strain, and the CC042 strain having 1000-fold higher CFUs than B6 susceptible strain. Strain CC046/Unc exhibited a new phenotype combination with opposite CFU levels between the two target organs studied, (CFUs at high level similar to B6 in the liver and at low level similar to 129 in the spleen). Our findings demonstrate that the host genetic diversity provided by the CC population enables to unravel new diverse phenotypes previously unseen in classical laboratory strains. These extreme and rare strains represent new models to study the pathophysiology of *Salmonella* infections and to explore how host genetic differences affect susceptibility.

The contrast between strains could be influenced by non-genetic factors such as the microbiota. However, most strains were bred in the same room under SPF conditions and the IV route of infection we used bypasses the intestinal phase and results in rapid septicemia, minimizing a potential influence of microbiota.

We identified two significant and one suggestive QTLs for spleen bacterial load as well as two suggestive QTLs for liver bacterial load. Despite a high and positive Pearson correlation coefficient between the two phenotypes (R^2^ = 0.88), no QTLs common to both organs were identified. In fact, none of the QTLs identified for spleen load was even close to significance for the liver, and reciprocally. Several factors may explain this finding. First, the correlation between the two traits may not be strong enough for a QTL primarily associated with one trait to be detected secondarily with the other trait. Second, the number of strains and the effect size of each QTL may be limiting. Finally, it is possible that bacterial proliferation in spleen and liver are under the control of different genes, and different mechanisms.

By using the CC reference population which includes three wild-derived founders, we expected to identify novel host genetic variants and mechanisms to infectious diseases. Previous studies identified various QTLs implicated in the differences in host immunity to infection with *Salmonella* Typhimurium: Immunity to Typhimurium-*Ity* [10, 19, 47, 48], Modifier of *Salmonella* Typhimurium Susceptibility-*Msts* [49], Susceptibility to *Salmonella* Typhimurium Antigens-*Ssta* [50, 51]. None of them localized in the same regions on Chr 8 and 10 that were identified in our study. Previous studies identified three QTLs for *Salmonella* susceptibility using crosses with susceptible wild-derived *Mus m. molossinus* MOLF/Ei strain [9, 47]. Likewise, we found that the two significant QTLs we mapped involved a contrast with one of the three wild strains, highlighting the importance of wild-derived founders’ contribution in the CC.

It is well known that the *Slc11a1* and *Tlr4* genes have major influences in the susceptibility to *Salmonella* of laboratory strains. B6 inbred strain is susceptible due to a single missense mutation in *Slc11a1* [12]. The broad critical interval for *Stsl3a* and *Stsl3b* suggestive QTLs on Chr 1 (70–100 Mb) contains *Slc11a1* (74.3 Mb). Interestingly, the CC042 strain that exhibits an extreme susceptibility phenotype has inherited a B6 susceptible haplotype at *Scl11a1* locus. However, this allele alone is not sufficient to explain the extreme phenotype of this strain as three other CC strains that inherited the same *Slc11a1* susceptible allele from B6 founder origin (CC0021/Unc, CC045/GeniUnc and C0061/GeniUnc) have 10 to 12-fold lower splenic bacterial loads than B6 (see Additional file 8: Figure S5). Another important gene involved in susceptibility to Gram-negative bacteria is *Tlr4*. Although no QTL was detected on Chr 4 where *Tlr4* is localized, we assessed the functionality of *Tlr4* in CC042. This strain inherited a wild-derived PWK/PhJ *Tlr4* haplotype which contains several missense mutations. We confirmed by LPS stimulation that this PWK/PhJ derived *Tlr4* allele is functional in CC042 strain. Moreover, three other CC strains inherited the same PWK/PhJ *Tlr4* haplotype (CC006/TauUnc, CC052/GeniUnc, CC061/GeniUnc, see Additional file 8: Figure S5) and have resistant to intermediate splenic bacterial loads (respectively 5.3, 4.34 and 4.94) which show that this allele is not associated with high susceptibility. These results emphasize that host genetic resistance to *Salmonella* Typhimurium is complex with many genes interacting. Major genes identified in classical laboratory strains may not have the same impact in a population harboring more genetic diversity.

In this study we used *Salmonella* Typhimurium strain SL1344. To confirm that our results are not specific to this bacterial strain, CC042 mice were infected with *S.* Typhimurium strain Keller. CC042 mice present the same degree of extreme susceptibility (1000 higher CFUs in spleen and liver, data not shown) as compared to B6 mice. This correlates with previous evidence in the literature that the same host susceptibility loci can be identified by different *S.* Typhimurium strains. *Msts* 1–4 loci were identified using *S.* Typhimurium C5 strain [49] and correspond to loci previously identified on Chr 1 (*Slc11a1*), Chr 6, Chr 11 (*Ity2*) and Chr 13 (*Ity13*) using *S.* Typhimurium Keller strain [9, 19, 47, 48].

The confidence intervals of the two major QTLs, *Stsl1* and *Stsl2* contain too many genes to directly point at likely candidate causal genes. In order to prioritize them, we used sequence variation information and merge analysis strategy [36] combined with gene expression, known function and phenotypes, to refine *Stsl1* and *Stsl2* QTLs intervals and identify candidate genes. Within *Stsl1* QTL, four genes are strong candidates based on known phenotypes: *Cul4a* (cullin 4A) deficient animal die in utero and *Cul4a* is essential for hematopoietic cell survival [52]; *Lamp1* (lysosomal-associated membrane protein 1) is highly expressed in macrophages and is involved in autophagy as well as protecting NK cell from degranulation-associated damage [53, 54]; *Mcf2l* (mcf.2 transforming sequence-like) targeted mutant mice (IMPC) have a decreased number of CD8-positive T cells; *Pcid2* (PCI domain containing 2) is essential for spleen development and regulation of B cell differentiation [55]. Within *Stsl2* QTL one gene is a high-potential candidate: *Slc35f1* targeted mutant have a decreased lactate dehydrogenase activity (IMPC, Phenotype MP:0005571), which may alter the pyruvate metabolism pathway in *Salmonella* [56].

Using computer simulation, it was reported that at least 500 strains were required to map a single additive QTL that explains 5% of the phenotypic variation [20]. However, such large numbers are not available since ~ 95% of CC strains became extinct during the inbreeding process, so that only 70 strains are distributed [57, 58]. In our study, we have shown that even a smaller number of CC strains (here, 35) can provide enough power to identify QTLs with genome-wide significance. We used our experimental data to estimate the minimum number of strains needed to identify the two major QTLs found with 35 strains. We found that 20 strains almost always missed them, while 30 strains were almost as successful as the full set of 35 at identifying *Stsl1*. This conclusion is dependent on the size of QTL effect.

## Conclusion

By exploring a broader genetic variation, the Collaborative Cross population has revealed novel loci of resistance to *Salmonella* Typhimurium. It also led to the identification of CC042 as an extremely susceptible strain. This study provides further example of the power of the CC resource to observe novel phenotypes and identify additional host genes controlling quantitative traits such as the susceptibility to infections. These results will further enhance our capacity to understand the complex host-bacteria interplay.

## Additional files

Additional file 1: **Table S1**. Individual organ bacterial loads at day 4 post-infection with *Salmonella* Typhimurium. Individual values for each animal tested are given: animal number (N), strain, alias (collected from UNC Systems Genetics), sex (Females | Males), spleen bacterial load as log_10_ *p*-value of CFUs per gram of spleen (log10.CFUs.g.Spleen), liver bacterial load as log_10_ p-value of CFUs per gram of liver (log10.CFUs.g.Liver) and experiment. NA: missing value. (XLSX 13 kb)

Additional file 2: **Figure S1**. QTLs associated with bacterial loads in spleen after *S.* Typhimurium infection in different subsets of CC strains. X-axis: genome location of each QTL *Stls1* and *Stls2* identified in Fig. 2; Y-axis: probability of detecting QTLs at different genomic significance (E < 0.5 in gray, E < 0.1 in orange, E < 0.05 in red and combined in blue). Genome-wide thresholds of association at E < 0.5, E < 0.1 and E < 0.05 significance levels of each test were determined by 200 permutation tests. Subsets of 15, 20, 25, 30 and 34 CC strains were tested. Within each subset, 500 random permutations were tested, except for subset of 34 CC, with only 35 possible permutations. (PDF 66 kb)

Additional file 3: **Figure S2**. Founder contributions and haplotype around *Stsl3* QTL on Chr 1. (A) Genome scan magnification for *Stsl3* QTL region (70–100 Mb on Chr 1). The mouse genome location is on the X-axis and significance (−log_10_(P)) values on the Y-axis, with genome-wide thresholds of association at E < 0.5, E < 0.1 and E < 0.05 levels indicated respectively by the gray, orange and red lines. Peak locations *Stsl3a* and *Stsl3b* (maximum value of –log_10_(P)) are marked by stars. (B) Founder contributions in the same magnified region. The peak location of *Stsl3a* is marked by a star. Each of the 8 founders is in a different color. The mouse genome location is on the X-axis and Y-axis shows the founder estimated effect on splenic bacterial load after *S.* Typhimurium infection. (C) Founder contributions at *Stsl3a* QTL peak (83.9 Mb). X-axis shows the different founder strains. Y-axis shows the estimated founder effect. No obvious contributions explain *Stsl3a* QTL, but B6 (grey) has the highest estimated impact of the 8 founders. (D) Founder contributions at *Stsl3b* QTL peak (79.2 Mb). There is no obvious founder contribution for *Stsl3b* QTL peak region. 129 (pink) has the highest estimated impact of the 8 founders while PWK (red) has the lowest estimate. (PDF 215 kb)

Additional file 4: **Figure S3**. Founder contributions and haplotype around *Stsl4* QTL on Chr 6. (A) Genome scan magnification for *Stsl4* QTL region (60–100 Mb on Chr 6). The mouse genome location is on the X-axis and significance (−log_10_(P)) values on the Y-axis, with genome-wide thresholds of association at E < 0.5, E < 0.1 and E < 0.05 levels indicated respectively by the gray, orange and red lines. Peak location (maximum value of –log_10_(P)) is marked by a star. (B) Founder contributions in the same magnified region. The peak location is marked by a star. Each of the 8 founders is in a different color. The mouse genome location is on the X-axis and Y-axis shows the founder estimated effect on splenic bacterial load after *S.* Typhimurium infection. (C) Founder contributions at *Stsl4* QTL peak (81.2 Mb). X-axis shows the different founder strains. Y-axis shows the estimated founder effect. No obvious contributions explain *Stsl4* QTL, but B6 has the lowest estimated impact while NZO/HILtJ and PWK/PhJ have the highest estimates. (PDF 160 kb)

Additional file 5: **Figure S4**. Founder contributions and haplotype around *Stsl5* QTL on Chr 17. (A) Genome scan magnification for *Stsl5* QTL region (75–95 Mb on Chr 17). The mouse genome location is on the X-axis and significance (−log_10_(P)) values on the Y-axis, with genome-wide thresholds of association at E < 0.5, E < 0.1 and E < 0.05 levels indicated respectively by the gray, orange and red lines. Peak location (maximum value of –log_10_(P)) is marked by a star. (B) Founder contributions in the same magnified region. The peak location is marked by a star. Each of the 8 founders is in a different color. The mouse genome location is on the X-axis and Y-axis shows the founder estimated effect on splenic bacterial load after *S.* Typhimurium infection. (C) Founder contributions at *Stsl5* QTL peak (84.8 Mb). X-axis shows the different founder strains. Y-axis shows the estimated founder effect. No obvious contributions explain *Stsl5* QTL, but B6 has the highest estimated impact while NOD/ShiLtJ has the lowest. (PDF 123 kb)

Additional file 6: **Table S2**. Genes remaining in *Stls1* interval post merge analysis. Gene symbol, start and end positions, name, high merged SNPs, expression in immune cell, cell-type major expression and Gene Ontology (GO) terms are given. Gene positions (build mm9), names as well as GO terms were collected from UCSC, MGI and ENSEMBL, while expression data were collected from Male/Female RNAseq of ImmGen. (XLSX 496 kb)

Additional file 7: **Table S3**. Genes remaining in *Stls2* interval post merge analysis. Gene symbol, start and end positions, name, high merged SNPs, expression in immune cell, cell-type major expression and Gene Ontology (GO) terms are given. Gene positions (build mm9), names as well as GO terms were collected from UCSC, MGI and ENSEMBL, while expression data were collected from Male/Female RNAseq of ImmGen. (XLSX 492 kb)

Additional file 8: **Figure S5**. CC strains carrying either *Tlr4 < PWK >* or *Slc11a1 < B6>*. Same data as on Fig. 1. Strains carrying *Slc11a1 < B6 >* susceptible allele are highlighted in red boxes. Strains carrying *Tlr4 < PWK >* allele are highlighted in blue circles. None of these alleles is associated with higher or lower bacterial loads in spleen or liver. (PDF 231 kb)

## Abbrevations

129: 129S2/SvPasCrl
B6: C57BL/6 J
CC: Collaborative Cross
CC042: CC042/GeniUnc
Chr: Chromosome
QTL: Quantitative Trait Locus
SDP: Strain Distribution Pattern
SNP: Single Nucleotide Polymorphism

## References

[1] LaRock DL, Chaudhary A, Miller SI (2015). Salmonellae interactions with host processes. Nat Rev Microbiol.

[2] Fàbrega A, Vila J (2013). Salmonella enterica serovar typhimurium skills to succeed in the host: virulence and regulation. Clin Microbiol Rev.

[3] de Jong HK, Parry CM, van der Poll T, Wiersinga WJ (2012). Host-pathogen interaction in invasive salmonellosis. PLoS Pathog.

[4] Majowicz SE, Musto J, Scallan E, Angulo FJ, Kirk M, O’Brien SJ (2010). The global burden of Nontyphoidal Salmonella gastroenteritis. Clin Infect Dis.

[5] Roy M-F, Malo D (2002). Genetic regulation of host responses to Salmonella infection in mice. Genes Immun.

[6] O’Brien AD, Taylor BA, Rosenstreich DL. Genetic control of natural resistance to Salmonella typhimurium in mice during the late phase of infection. J Immunol. 1984;133 6386984

[7] Santos RL, Zhang S, Tsolis RM, Kingsley RA, Adams LG, Bäumler AJ. Animal models of Salmonella infections: enteritis versus typhoid fever. Microbes Infect. 3:1335–44. 10.1016/s1286-4579(01)01495-2 11755423

[8] Robson HG, Vas SI (1972). Resistance of inbred mice to Salmonella typhimurium. J Infect Dis.

[9] Sebastiani G, Olien L, Gauthier S, Skamene E, Morgan K, Gros P (1998). Mapping of genetic modulators of natural resistance to infection with Salmonella typhimurium in wild-derived mice. Genomics.

[10] Roy MF, Riendeau N, Bedard C, Helie P, Min-Oo G, Turcotte K (2007). Pyruvate kinase deficiency confers susceptibility to Salmonella typhimurium infection in mice. J Exp Med.

[11] Richer E, Yuki KE, Dauphinee SM, Larivière L, Paquet M, Malo D (2011). Impact of Usp18 and IFN signaling in Salmonella-induced typhlitis. Genes Immun.

[12] Vidal S, Tremblay ML, Govoni G, Gauthier S, Sebastiani G, Malo D (1995). The Ity/Lsh/Bcg locus: natural resistance to infection with intracellular parasites is abrogated by disruption of the Nramp1 gene. J Exp Med.

[13] Poltorak A, He X, Smirnova I, Liu MY, Van Huffel C, Du X (1998). Defective LPS signaling in C3H/HeJ and C57BL/10ScCr mice: mutations in Tlr4 gene. Science.

[14] Rawlings DJ, Saffran DC, Tsukada S, Largaespada DA, Grimaldi JC, Cohen L (1993). Mutation of unique region of Bruton’s tyrosine kinase in immunodeficient XID mice. Science.

[15] Qureshi ST, Lariviere L, Leveque G, Clermont S, Moore KJ, Gros P (1999). Endotoxin-tolerant mice have mutations in toll-like receptor 4 (Tlr4). J Exp Med.

[16] Hu J, Bumstead N, Barrow P, Sebastiani G, Olien L, Morgan K (1997). Resistance to salmonellosis in the chicken is linked to NRAMP1 and TNC. Genome Res.

[17] Leveque G, Forgetta V, Morroll S, Smith AL, Bumstead N, Barrow P (2003). Allelic variation in TLR4 is linked to susceptibility to Salmonella enterica serovar typhimurium infection in chickens. Infect Immun.

[18] Vidal SM, Malo D, Marquis JF, Gros P (2008). Forward genetic dissection of immunity to infection in the mouse. Annu Rev Immunol.

[19] Roy MF, Riendeau N, Loredo-Osti JC, Malo D (2006). Complexity in the host response to Salmonella typhimurium infection in AcB and BcA recombinant congenic strains. Genes Immun.

[20] Flint J, Valdar W, Shifman S, Mott R (2005). Strategies for mapping and cloning quantitative trait genes in rodents. Nat Rev Genet.

[21] Churchill GA, Airey DC, Allayee H, Angel JM, Attie AD, Beatty J (2004). The collaborative cross, a community resource for the genetic analysis of complex traits. Nat Genet.

[22] Morgan AP, Welsh CE (2015). Informatics resources for the collaborative cross and related mouse populations. Mamm Genome.

[23] Threadgill DW, Miller DR, G a C, de Villena FP-M (2011). The collaborative cross: a recombinant inbred mouse population for the systems genetic era. ILAR J.

[24] Roberts A, Pardo-Manuel de Villena F, Wang W, McMillan L, Threadgill DW. The polymorphism architecture of mouse genetic resources elucidated using genome-wide resequencing data: implications for QTL discovery and systems genetics. Mamm Genome 2007;18:473–481. 10.1007/s00335-007-9045-1 PMC1998888 17674098

[25] Aylor DL, Valdar W, Foulds-Mathes W, Buus RJ, Verdugo R a, Baric RS, et al. Genetic analysis of complex traits in the emerging collaborative cross. Genome Res 2011;21:1213–1222. 10.1101/gr.111310.110 PMC3149489 21406540

[26] Philip VM, Sokoloff G, Ackert-Bicknell CL, Striz M, Branstetter L, Beckmann MA (2011). Genetic analysis in the collaborative cross breeding population. Genome Res.

[27] Welsh CE, Miller DR, Manly KF, Wang J, McMillan L, Morahan G (2012). Status and access to the collaborative cross population. Mamm Genome.

[28] Iraqi FA, Churchill G, Mott R (2008). The collaborative cross, developing a resource for mammalian systems genetics: a status report of the Wellcome Trust cohort. Mamm Genome.

[29] Morahan G, Balmer L, Monley D (2008). Establishment of “the gene mine”: a resource for rapid identification of complex trait genes. Mamm Genome.

[30] Chesler EJ, Miller DR, Branstetter LR, Galloway LD, Jackson BL, Philip VM (2008). The collaborative cross at oak Ridge National Laboratory: developing a powerful resource for systems genetics. Mamm Genome.

[31] Collaborative Cross Consortium CC (2012). The genome architecture of the collaborative cross mouse genetic reference population. Genetics.

[32] Yang H, Ding Y, Hutchins LN, Szatkiewicz J, Bell TA, Paigen BJ (2009). A customized and versatile high-density genotyping array for the mouse. Nat Methods.

[33] Mott R, Talbot CJ, Turri MG, Collins AC, Flint J (2000). A method for fine mapping quantitative trait loci in outbred animal stocks. Proc Natl Acad Sci U S A.

[34] Durrant C, Tayem H, Yalcin B, Cleak J, Goodstadt L (2011). Pardo-Manuel de Villena F, et al. collaborative cross mice and their power to map host susceptibility to aspergillus fumigatus infection. Genome Res.

[35] Durrant C, Mott R (2010). Bayesian quantitative trait locus mapping using inferred haplotypes. Genetics.

[36] Yalcin B, Flint J, Mott R. Using Progenitor Strain Information to Identify Quantitative Trait Nucleotides in Outbred Mice. Genetics. 2005;681 October:673–681. 10.1534/genetics.104.028902 PMC1456780 16085706

[37] MGI-Mouse Genome Informatics-The international database resource for the laboratory mouse. http://www.informatics.jax.org/. Accessed 15 Jun 2017.

[38] Carlson M, Maintainer B. TxDb.Mmusculus.UCSC.mm9.knownGene: Annotation package for TxDb object(s). 2015. R package version 3.2.2. DOI10.18129/B9.bioc.TxDb.Mmusculus.UCSC.mm9.knownGene

[39] Immunological Genome Project-ImmGen. https://www.immgen.org/. Accessed 15 Jun 2017.

[40] Ensembl genome browser. http://www.ensembl.org/index.html. Accessed 15 Jun 2017.

[41] IMPC | International Mouse Phenotyping Consortium. http://www.mousephenotype.org/. Accessed 15 Jun 2017.

[42] Vered K, Durrant C, Mott R, Iraqi FA (2014). Susceptibility to klebsiella pneumonaie infection in collaborative cross mice is a complex trait controlled by at least three loci acting at different time points. BMC Genomics.

[43] Sanger Mouse SnpViewer. http://www.sanger.ac.uk/sanger/Mouse_SnpViewer/rel-1505. Accessed 15 Jun 2017.

[44] O’Brien AD, Rosenstreich DL, Scher I, Campbell GH, MacDermott RP, Formal SB. Genetic control of susceptibility to Salmonella typhimurium in mice: role of the LPS gene. J Immunol. 1980;124 6985638

[45] Hoshino K, Takeuchi O, Kawai T, Sanjo H, Ogawa T, Takeda Y (1999). Cutting edge: toll-like receptor 4 (TLR4)-deficient mice are hyporesponsive to lipopolysaccharide: evidence for TLR4 as the Lps gene product. J Immunol.

[46] Yamakawa T, Tanaka SI, Yamakawa Y, Isoda F, Kawamoto S, Fukushima J (1996). Genetic control of in vivo tumor necrosis factor production in mice. Clin Immunol Immunopathol.

[47] Sebastiani G, Blais V, Sancho V, Vogel SN, Stevenson MM, Gros P (2002). Host immune response to Salmonella enterica serovar typhimurium infection in mice derived from wild strains. Infect Immun.

[48] Sancho-Shimizu V, Khan R, Mostowy S, Larivière L, Wilkinson R, Riendeau N (2007). Molecular genetic analysis of two loci (Ity2 and Ity3) involved in the host response to infection with Salmonella typhimurium using congenic mice and expression profiling. Genetics.

[49] Borrego A, Peters LC, Jensen JR, Ribeiro OG, Koury Cabrera WH, Starobinas N (2006). Genetic determinants of acute inflammation regulate Salmonella infection and modulate Slc11a1 gene (formerly Nramp1) effects in selected mouse lines. Microbes Infect.

[50] de Souza CM, Morel L, Cabrera WH, Starobinas N, Ribeiro OG, Siqueira M (2004). Quantitative trait loci in chromosomes 3, 8, and 9 regulate antibody production against Salmonella flagellar antigens in the mouse. Mamm Genome.

[51] Trezena AG, Souza CM, Borrego A, Massa S, Siqueira M, De Franco M (2002). Co-localization of quantitative trait loci regulating resistance to Salmonella typhimurium infection and specific antibody production phenotypes. Microbes Infect.

[52] Waning DL, Li B, Jia N, Naaldijk Y, Goebel WS, Hogeneseh H (2008). Cul4A is required for hematopoietic cell viability and its deficiency leads to apoptosis. Blood.

[53] Saftig P, Klumperman J (2009). Lysosome biogenesis and lysosomal membrane proteins: trafficking meets function. Nat Rev Mol Cell Biol.

[54] Cohnen A, Chiang SC, Stojanovic A, Schmidt H, Claus M, Saftig P (2013). Surface CD107a / LAMP-1 protects natural killer cells from degranulation-associated damage. Immunobiology.

[55] Nakaya T, Kuwahara K, Ohta K, Kitabatake M, Toda T, Takeda N (2010). Critical role of Pcid2 in B cell survival through the regulation of MAD2 expression. J Immunol.

[56] Abernathy J, Corkill C, Hinojosa C, Li X, Zhou H (2013). Deletions in the pyruvate pathway of Salmonella typhimurium alter SPI1-mediated gene expression and infectivity. J Anim Sci Biotechnol.

[57] Shorter JR, Odet F, Aylor DL, Pan W, Kao C-Y, Fu C-P (2017). Male infertility is responsible for nearly half of the extinction observed in the mouse collaborative cross. Genetics.

[58] Srivastava A, Morgan AP, Najarian ML, Sarsani VK, Sigmon JS, Shorter JR (2017). Genomes of the mouse collaborative cross. Genetics.

