# Supplementary figures and images for: Identification of new loci involved in the host susceptibility to Salmonella Typhimurium in collaborative cross mice
Source: BMC Genomics. 2018 Apr 27;19:303. doi: 10.1186/s12864-018-4667-0 (PMC5923191; doi:10.1186/s12864-018-4667-0)

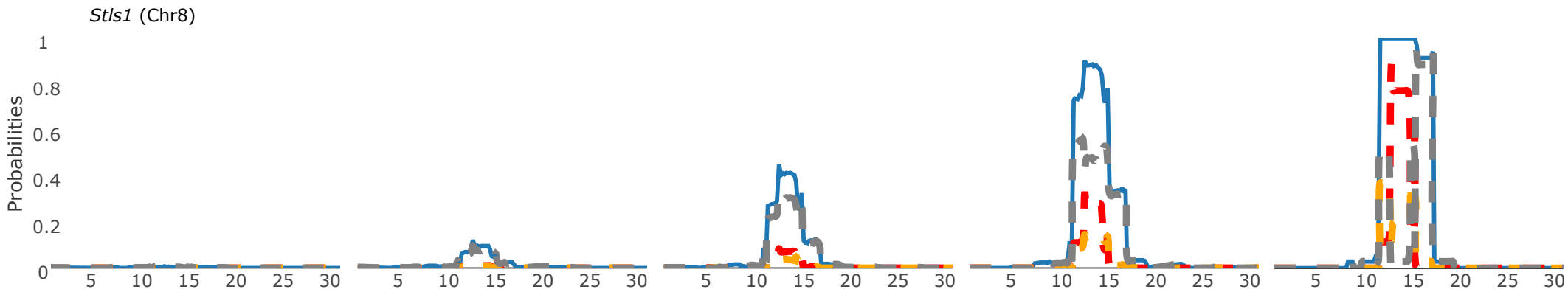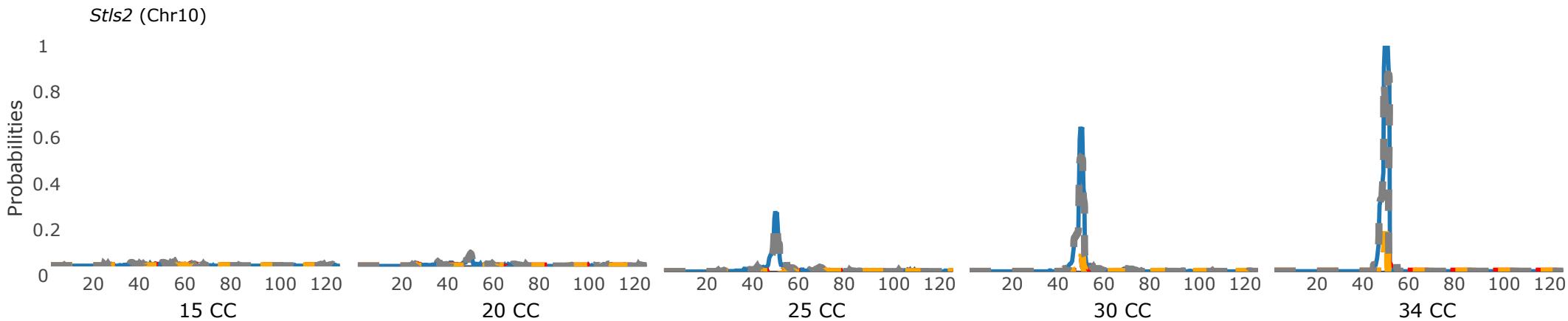

Supplement: Supplementary file 2 — Figure S1. QTLs associated with bacterial loads in spleen after S. Typhimurium infection in different subsets of CC strains. X-axis: genome location of each QTL Stls1 and Stls2 identified in Fig. 2; Y-axis: probability of detecting QTLs at different genomic significance (E < 0.5 in gray, E < 0.1 in orange, E < 0.05 in red and combined in blue). Genome-wide thresholds of association at E < 0.5, E < 0.1 and E < 0.05 significance levels of each test were determined by 200 permutation tests. Subsets of 15, 20, 25, 30 and 34 CC strains were tested. Within each subset, 500 random permutations were tested, except for subset of 34 CC, with only 35 possible permutations. (PDF 66 kb) [file 12864_2018_4667_MOESM2_ESM.pdf]

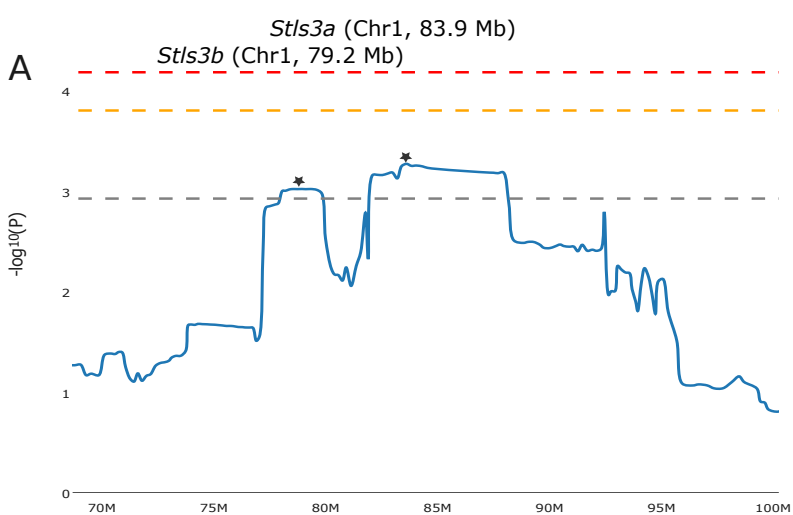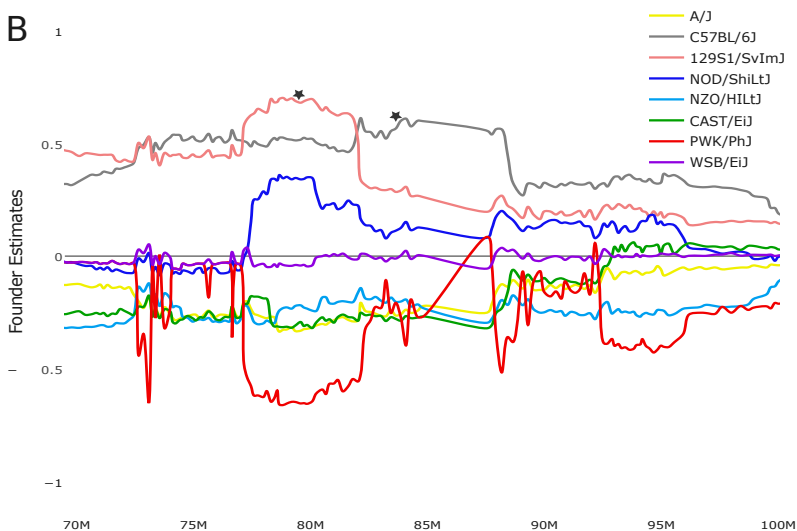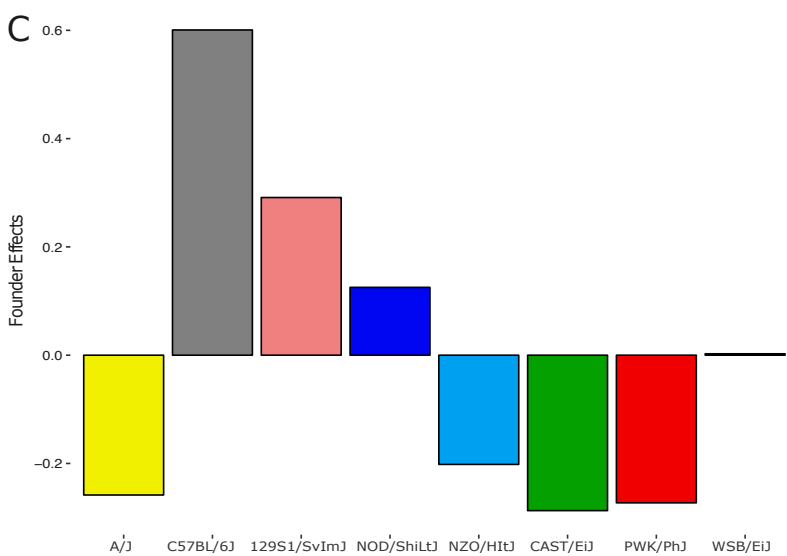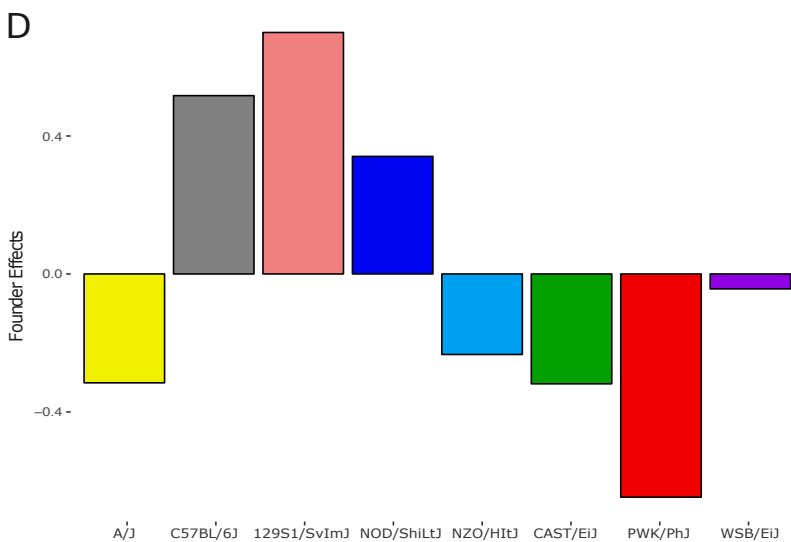

Supplement: Supplementary file 3 — Figure S2. Founder contributions and haplotype around Stsl3 QTL on Chr 1. (A) Genome scan magnification for Stsl3 QTL region (70–100 Mb on Chr 1). The mouse genome location is on the X-axis and significance (−log10(P)) values on the Y-axis, with genome-wide thresholds of association at E < 0.5, E < 0.1 and E < 0.05 levels indicated respectively by the gray, orange and red lines. Peak locations Stsl3a and Stsl3b (maximum value of –log10(P)) are marked by stars. (B) Founder contributions in the same magnified region. The peak location of Stsl3a is marked by a star. Each of the 8 founders is in a different color. The mouse genome location is on the X-axis and Y-axis shows the founder estimated effect on splenic bacterial load after S. Typhimurium infection. (C) Founder contributions at Stsl3a QTL peak (83.9 Mb). X-axis shows the different founder strains. Y-axis shows the estimated founder effect. No obvious contributions explain Stsl3a QTL, but B6 (grey) has the highest estimated impact of the 8 founders. (D) Founder contributions at Stsl3b QTL peak (79.2 Mb). There is no obvious founder contribution for Stsl3b QTL peak region. 129 (pink) has the highest estimated impact of the 8 founders while PWK (red) has the lowest estimate. (PDF 215 kb) [file 12864_2018_4667_MOESM3_ESM.pdf]

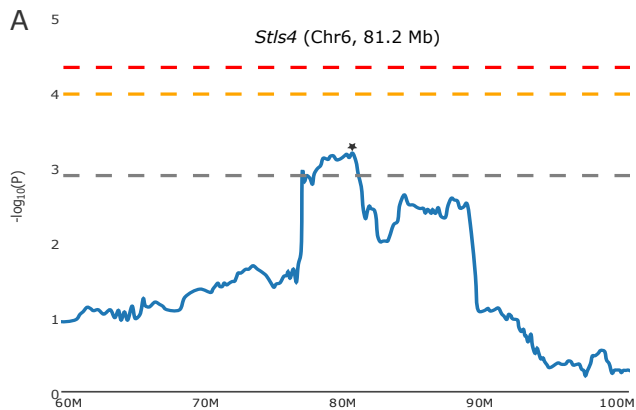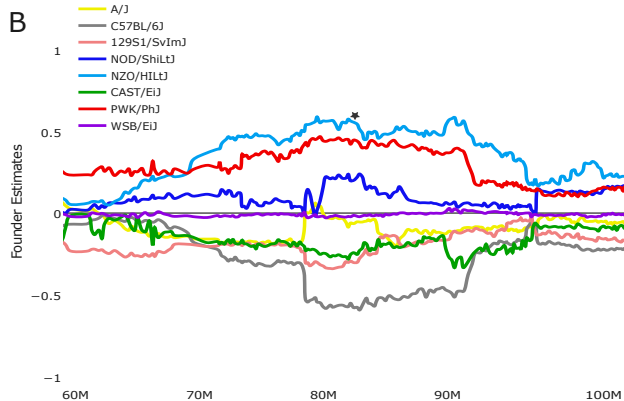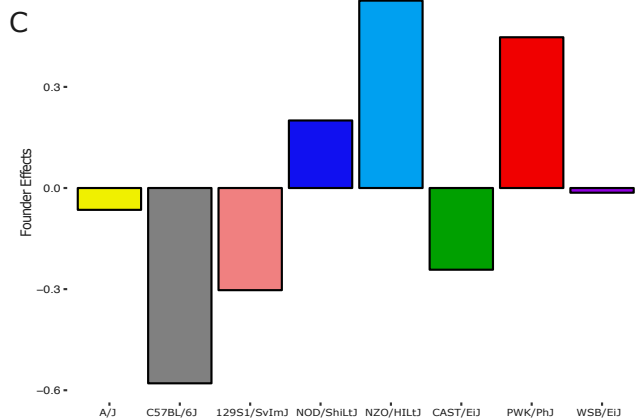

Supplement: Supplementary file 4 — Figure S3. Founder contributions and haplotype around Stsl4 QTL on Chr 6. (A) Genome scan magnification for Stsl4 QTL region (60–100 Mb on Chr 6). The mouse genome location is on the X-axis and significance (−log10(P)) values on the Y-axis, with genome-wide thresholds of association at E < 0.5, E < 0.1 and E < 0.05 levels indicated respectively by the gray, orange and red lines. Peak location (maximum value of –log10(P)) is marked by a star. (B) Founder contributions in the same magnified region. The peak location is marked by a star. Each of the 8 founders is in a different color. The mouse genome location is on the X-axis and Y-axis shows the founder estimated effect on splenic bacterial load after S. Typhimurium infection. (C) Founder contributions at Stsl4 QTL peak (81.2 Mb). X-axis shows the different founder strains. Y-axis shows the estimated founder effect. No obvious contributions explain Stsl4 QTL, but B6 has the lowest estimated impact while NZO/HILtJ and PWK/PhJ have the highest estimates. (PDF 160 kb) [file 12864_2018_4667_MOESM4_ESM.pdf]

**A**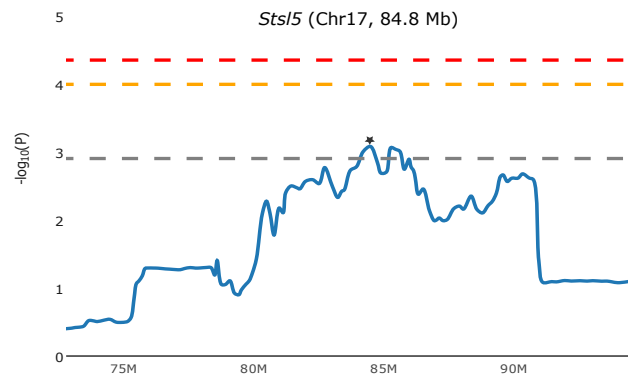**B**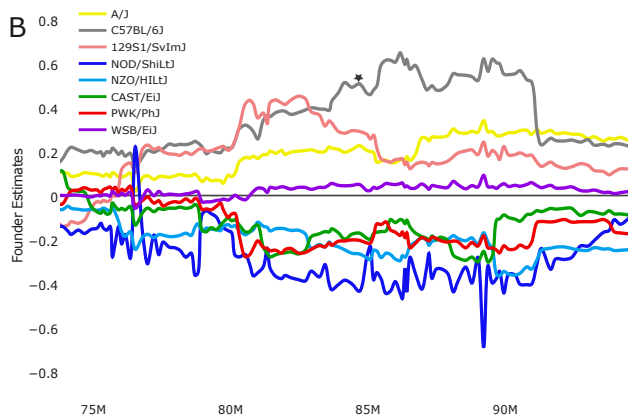**C**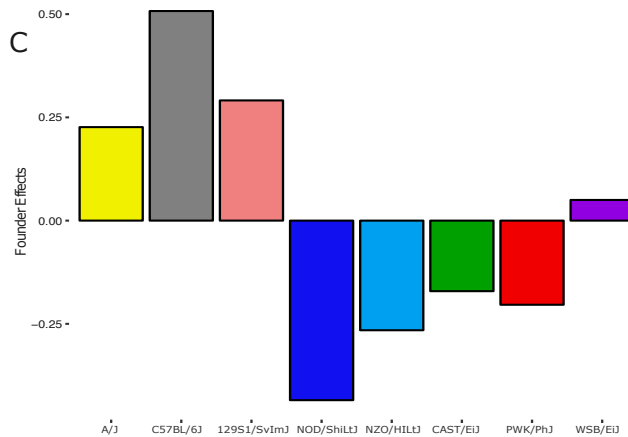

Supplement: Supplementary file 5 — Figure S4. Founder contributions and haplotype around Stsl5 QTL on Chr 17. (A) Genome scan magnification for Stsl5 QTL region (75–95 Mb on Chr 17). The mouse genome location is on the X-axis and significance (−log10(P)) values on the Y-axis, with genome-wide thresholds of association at E < 0.5, E < 0.1 and E < 0.05 levels indicated respectively by the gray, orange and red lines. Peak location (maximum value of –log10(P)) is marked by a star. (B) Founder contributions in the same magnified region. The peak location is marked by a star. Each of the 8 founders is in a different color. The mouse genome location is on the X-axis and Y-axis shows the founder estimated effect on splenic bacterial load after S. Typhimurium infection. (C) Founder contributions at Stsl5 QTL peak (84.8 Mb). X-axis shows the different founder strains. Y-axis shows the estimated founder effect. No obvious contributions explain Stsl5 QTL, but B6 has the highest estimated impact while NOD/ShiLtJ has the lowest. (PDF 123 kb) [file 12864_2018_4667_MOESM5_ESM.pdf]
