# Supplementary material for: Identification of new loci involved in the host susceptibility to Salmonella Typhimurium in collaborative cross mice
Source: BMC Genomics. 2018 Apr 27;19:303. doi: 10.1186/s12864-018-4667-0 (PMC5923191; doi:10.1186/s12864-018-4667-0)
Supplement: Supplementary file 8 — Figure S5. CC strains carrying either Tlr4 < PWK > or Slc11a1 < B6>. Same data as on Fig. 1. Strains carrying Slc11a1 < B6 > susceptible allele are highlighted in red boxes. Strains carrying Tlr4 < PWK > allele are highlighted in blue circles. None of these alleles is associated with higher or lower bacterial loads in spleen or liver. (PDF 231 kb) [file 12864_2018_4667_MOESM8_ESM.pdf]

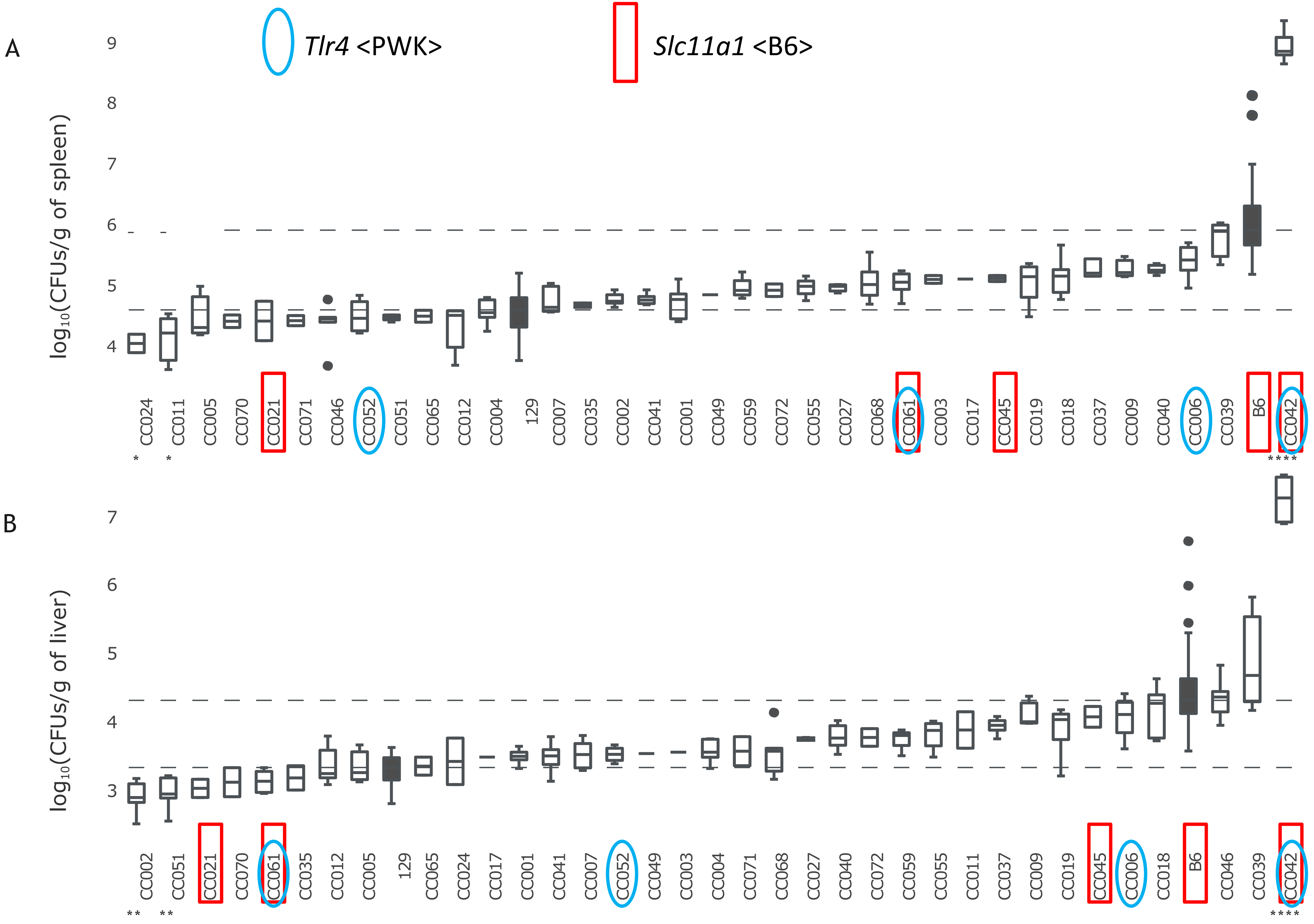

A
B
Tlr4 <PWK>
Slc11a1 <B6>
log10(CFUs/g of spleen)
log10(CFUs/g of liver)
CC024 CC011 CC005 CC070 CC021 CC071 CC046 CC052 CC051 CC065 CC012 CC004 129 CC007 CC035 CC002 CC041 CC001 CC049 CC059 CC072 CC055 CC027 CC068 CC061 CC003 CC017 CC045 CC019 CC018 CC037 CC009 CC040 CC006 CC039 B6 CC042
CC002 CC051 CC021 CC070 CC061 CC035 CC012 CC005 129 CC065 CC024 CC017 CC001 CC041 CC007 CC052 CC049 CC003 CC004 CC071 CC068 CC027 CC040 CC072 CC059 CC055 CC011 CC037 CC009 CC019 CC045 CC006 CC018 B6 CC046 CC039 CC042
